# A novel radial water tread maze tracks age-related cognitive decline in mice

**DOI:** 10.3402/pba.v3i0.20679

**Published:** 2013-10-04

**Authors:** Christina Pettan-Brewer, Dylan V. Touch, Jesse C. Wiley, Heather C. Hopkins, Peter S. Rabinovitch, Warren C. Ladiges

**Affiliations:** Department of Comparative Medicine, University of Washington, Seattle, WA, USA

**Keywords:** memory impairment, aging, water tread radial maze, mouse cognition

## Abstract

There is currently no treatment and cure for age-related dementia and cognitive impairment in humans. Mice suffer from age-related cognitive decline just as people do, but assessment is challenging because of cumbersome and at times stressful performance tasks. We developed a novel radial water tread (RWT) maze and tested male C57BL/6 (B6) and C57BL/6 x Balb/c F1 (CB6F1) mice at ages 4, 12, 20, and 28 months. B6 mice showed a consistent learning experience and memory retention that gradually decreased with age. CB6F1 mice showed a moderate learning experience in the 4 and 12 month groups, which was not evident in the 20 and 28 month groups. In conclusion, CB6F1 mice showed more severe age-related cognitive impairment compared to B6 mice and might be a suitable model for intervention studies. In addition, the RWT maze has a number of operational advantages compared to currently accepted tasks and can be used to assess age-related cognition impairment in B6 and CB6F1 mice as early as 12 months of age.

Decline in cognition is one of the major factors affecting quality of life in humans with increasing age. Memory impairment and dementia are assessed by a number of well-established procedures. Mice are used as models for aging research and suffer from age-related cognitive decline just as people do. However, assessment is challenging because of cumbersome and at times stressful testing conditions. Swimming is aversive to mice and is the basis for the classic Morris water maze cognitive memory task described in many research publications (1). The swim test has been the gold standard for assessment of cognitive function in mice but is difficult to set this up in specific pathogen-free facilities. The natural tendency of mice to escape bright light exposure is the basis for the Barnes radial maze behavioral test (2). The Barnes maze requires visual cues to optimize the cognitive performance. We wanted to combine the aversive qualities of both tasks in a paradigm that had minimal interpretive disadvantages. We therefore developed a novel radial maze using a shallow layer of water requiring mice to wade to a dark escape hole containing a warm environment, food, and drinking water as a positive reinforcement. Using this radial water tread (RWT) maze paradigm, we have shown that mice expressing mitochondrial catalase have improved cognition (3), and APP/Psen1 mutant mice (model for Alzheimer's disease) treated with phenylbutyrate maintain normal cognition with age compared to untreated mutant littermates that experience a severe decrease in cognitive ability with age (4). In this report, we describe the application of this novel cognitive paradigm in assessing the learning and memory capacity of two genetic wild type strains of mice at 4, 12, 20, and 28 months of age.

## Material and methods

### Experimental subjects

C57BL/6 (B6) (*N*=93) and Balb/c x C57BL/6 F1 (CB6F1) (*N*=93) male mice were obtained from the NIA mouse contract colony (Charles River, USA) at age groups of 4, 12, 20, and 28 months. Mice were housed at the animal research facility at the University of Washington and provided free access to a standard rodent diet (PicoLab^®^ Rodent Diet 20, Lab Diet, USA), water *ad libitum*, and acclimated for one week prior to testing. Animals were maintained throughout the study in a controlled environment (71F and 35% humidity) and a 12 h light/dark cycle, with behavioral tests conducted during the 12 h light cycle. All procedures were performed in accordance with protocols approved by the Animal Care and Use Committee at the University of Washington.

### Cognitive assessment

**Fig. 1:**
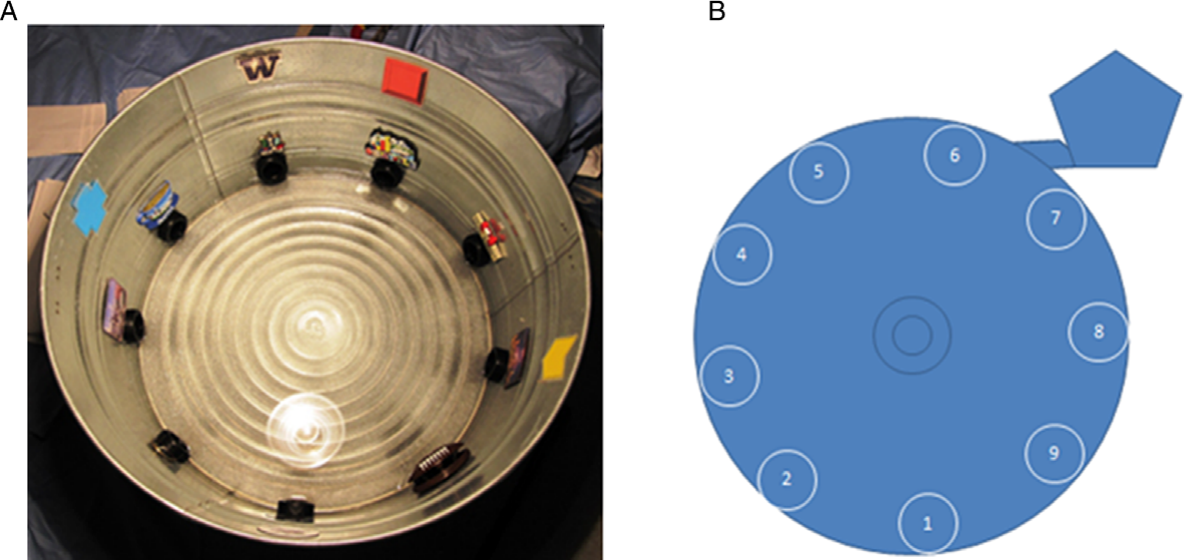
The radial water tread (RWT) maze is a low-cost, low-maintenance apparatus that is efficient to operate. The apparatus consists of a 30-inch circular galvanized enclosure with nine holes placed in the sides at regular intervals, as shown in (A) a photo and (B) a schematic drawing. The holes are rimmed with plastic pipe fittings. Eight of the holes are blocked and serve as decoys. One of the holes leads to a dark, escape ‘safe box’ hosting a comforting heating pad, food, and gel. All holes extend outward similar distances before either terminating or bending at a 90**°** angle to prevent direct visual determination of the actual escape route. The tub is filled with about one inch of water pre-warmed to room temperature, and a bright light is positioned directly over the entire apparatus to provide the escape incentives.

Mice were introduced into an approximately 30 inch circular galvanized metal enclosure with nine holes in the sides at regular intervals (Fig. 1). One of the holes led to a dark ‘safe box’ with a heating pad, food, and gel. The escape route and the decoy holes extended back to similar distances before either terminating, or bending at a 90**°** angle to prevent direct visual determination of the escape route. A bright light was placed overhead, and about 1 inch of room-temperature fresh water was added to the tank. The mice were introduced into the center of the enclosure, which contained unique visual images and objects along the sides, to function as spatial cues for the animal. The mice were then required to find the escape route from the enclosure. The maze was always placed in the same location in the room and the experimenter was positioned in the same location and side during the entire experiment. The water in the tank was changed and the tank was cleaned and sterilized between each cage to ensure consistency of environmental conditions. The animal was allowed to search for the correct escape exit for a 3 min period, after which time, the animal was hand-guided to the correct escape route in the first training days. Once the animal found the escape route, it was allowed to remain in the heated ‘safe box’ for 2 min prior to engaging in the next training trial. Mice were excluded from the group experiments if there were no successful escapes within the first two days, which usually reflected a general lack of antipathy for the task conditions, and hence no motivation to perform the task. If the animals placed the anterior portion of their body into a false escape route and did not voluntarily re-initiate exploration behavior within 10 s, the animal was gently placed back in the center of the tank.

The animals were given three trials per training day during a period known as the acquisition phase, which ran for 4 days. On day 5 and day 12, a probe trial was performed and the target exit was changed to a different location. The maze was rotated and re-adjusted so that the holes and the internal visual cues were still in the same positions as for the training days. Thus, we employed a four-day training period followed by the test days (probe trials) for shorter- and longer-term memory evaluations (day 5 and day 12, respectively).

### Statistical analysis

Numerical data were calculated using the Student's *t*-distribution and calculation of *p*-values.

## Results and discussion

**Fig. 2:**
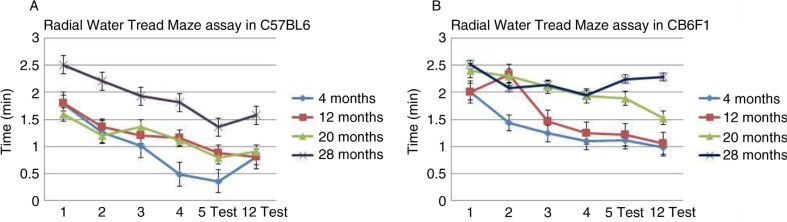
Learning and memory were moderately impaired with increasing age in B6 mice, but impairment was much more severe in CB6F1 mice with age. The data in Fig. 2 represent the average latency times of three trials each day during the acquisition phase (learning) and the day 5 and day 12 probe days (for shorter- and longer-term memory assessment, respectively). All mice were males with 93 animals for each genotype and a range of 20–23 mice for each age cohort. (A) C57BL/6 (B6) mice demonstrated exquisite learning and shorter- and longer-term memory retention at 4 months of age. Latency times increased with increasing age. *P*-values were ≤0.001. (B) CB6F1 mice showed no significant learning ability and no memory retention at 28 months of age. The 4 and 12 month age groups did have decreased latency times for the acquisition and probes trials, suggesting the strain has the ability to learn and retain memory at a young age. *P*-values were ≤0.001.

B6 mice showed a consistent learning experience that decreased with each age group (Fig. 2A). The average latency times during the four-day acquisition phase were 1.6–0.8 min for the 4, 12, and 20 month age groups and 2.1–1.5 min in the 28 month age group. During the probe trial on day 5, there was a significant difference in memory between the 4 and 28 month groups, with average latency times of 0.2 and 1.2 min, respectively. Average latency times for the day 12 probe trial were 0.2 min for the 4 and 12 month groups, and 0.8 and 1.2 min for the 20 and 28 month groups. Thus, we showed that latency times for B6 mice were significantly different between the younger and older age groups in the acquisition phase as well as during the probe trials on days 5 and 12 with *p*-values significant at <0.001 for all data sets.

CB6F1 mice at younger ages showed a moderate acquired learning experience with average latency times of 2.0–0.9 min in the 4 and 12 month groups in the acquisition phase (Fig. 2B). However, learning was not evident in the 20 and 28 month groups with average latency times of 2.4–1.8 min. During the probe trial on day 5, memory retention was evident in the two younger age groups with average latency times of 0.5 and 0.9 min, respectively. The 28 month group had no evidence of memory retention. The day 12 probe trial for CB6F1 mice showed similar findings as the probe trial on day 5, with latency times of 0.3 and 0.33 min for the two younger groups, respectively, and no evidence of memory retention in the 28 month group. Data sets were significant at *p*<0.001.

Our aim was to establish a model of age-related memory decline in different mouse strains using a novel cognitive paradigm, the radial water tread (RWT) maze. The RWT maze bears some resemblance to the Morris water maze and the Barnes maze spatial memory tests. It was used because it is easy to set up and administer and limits the exposure of mice to sources of infection, and with less dependence on limited exercise tolerance (swimming) of old mice. Our data showing significant learning and memory impairment at old ages further validate the RWT maze as a useful screening paradigm for cognitive assessment in mice.

B6 mice performed well in the RWT maze similar to previous reports in other spatial memory tasks. The data suggest that they were rapid learners during their young and adult life as has been previously reported (5, 6). On the other hand, our data suggest that CB6F1 mice were much less efficient at learning than B6 mice with age and had very little memory capacity at old age. Balb/c mice have been reported to show no apparent improvement during the acquisition learning phase (7), which is similar to our findings in old CB6F1 mice. Young CB6F1 mice showed some impairment in cognitive ability while old CB6F1 mice were more severely impaired, suggesting that this strain might be useful to investigate intervention of age-related memory decline.

An issue with cognition testing of old animals is whether existing disease conditions or impairments effecting mobility or sensory acuity might affect latency times. We feel confident that the data generated with the RWT maze are relevant because open field activity showed that CB6F1 mice, with extensive learning and memory impairments, were actually more active than B6 mice, especially at older ages, and they performed equally well on the rotarod apparatus. In addition, there was no difference in severity of cataracts with age in the two genotypes (manuscript in preparation). Therefore, it appears that these age-related physiological conditions did not skew the data generated by the RWT maze task.

In conclusion, there is currently still no treatment and cure for age-related dementia and cognitive impairment in humans, largely because the pathogenesis has not been fully understood. Mice are ideal for studying mechanisms of age-related memory impairment if the correct strain is selected. We emphasize the importance of using an efficient and productive cognitive assessment paradigm and suggest that the RWT maze has a number of operational advantages compared to currently accepted tasks.
